# Postoperative complications after successful primary rhegmatogenous retinal detachment repair

**DOI:** 10.1186/s12886-023-02824-5

**Published:** 2023-02-24

**Authors:** Lorenzo Motta, Rino Frisina, Matteo Ripa, Irene Gius, Angelo Greggio, Luigi Tozzi, Gabriella De Salvo, Alessandro Meduri

**Affiliations:** 1 Department of Ophthalmology, William Harvey Hospital, East Kent Hospitals University NHS Foundation Trust, Ashford, UK; 2 Department of Guglielmo da Saliceto Hospital, Ophthalmology Unit of Surgery, Piacenza, Italy; 3 Ophthalmology Unit, Fondazione Policlinico Universitario A. Gemelli IRCCS”, Rome, Italy; 4 Catholic University “Sacro Cuore”, Rome, Italy; 5 Ophthalmology Department, University of Padova, Padova, Italy; 6 Ophthalmology department, San Martino Hospital, Belluno, Italy; 7 Eye Unit, University Hospital Southampton NHS Foundation Trust, Southampton, UK; 8 Department of Biomedical Sciences, Eye Clinic, University of Messina, Messina, Italy

**Keywords:** Cystoid macular edema, Epiretinal membrane, Internal limiting membrane, Pars plana vitrectomy, Rhegmatogenous retinal detachment, Scleral buckling

## Abstract

**Background:**

To evaluate the incidence and risk factors for cystoid macular edema (CME) and epiretinal membrane (ERM) development after surgery for primary rhegmatogenous retinal detachment (RRD).

**Methods:**

Retrospective observational cohort study involving 62 consecutive patients with primary RRD who underwent RRD repair with either scleral buckling (SB) or pars plana vitrectomy (PPV). SB was used in young phakic patients without posterior vitreous detachment (PVD), high myopic patients, and RRD associated with either anterior or inferior retinal tears. PPV was preferred over SB in pseudophakic patients or those with media opacity and posterior breaks that precluded the SB approach. After surgery, the macular changes, including CME and ERM development, were evaluated 3 and 6 months postoperatively. Phacoemulsification and intraocular lens (IOL) implantation were performed in phakic patients where media opacity or lens bulging did not allow the surgeon to perform surgical maneuvers. The inner limiting membrane (ILM) peeling was randomly performed in the macula-off and the macula-on RRD “pending foveal detachment” subgroup.

**Results:**

Sixty-two eyes affected by RRD who underwent SB or PPV were enrolled. CME occurred in 33.3% of the PPV group regardless of the ERM formation. No CME cases were found in the SB group. Macula-off RRD increased the risk of CME by odds ratio (OR) = 4.3 times compared to macula-on RRD regardless of the surgical procedure (*p* = 0.04). Macula-off status increased the risk of CME of OR = 1.73 times compared to macula-on in the PPV subgroup (*p* = 0.4). Combined cataract surgery and PPV increased the risk of CME by OR = 3.3 times (*p* = 0.16) compared to PPV alone, and ILM peeling increased the risk of postoperative CME by OR = 1.8 times (*p* = 0.37). ERM occurred in 28% of patients who did not undergo ILM peeling, and 29.42% of those who underwent ILM peeling developed ERM (*p* = 0.6).

**Conclusions:**

The risk of postoperative CME was higher in patients with macula-off than in macula-on RRD and in those with macula-off RRD who underwent PPV. The SB would be advisable in patients with RRD sparing the macula. Furthermore, despite having several advantages, the combined phacoemulsification plus IOL implantation and PPV highly increased the risk of postoperative CME.

## Background

Primary rhegmatogenous retinal detachment (RRD) repair leads to satisfactory anatomical success with a low failure rate, regardless of the surgical approach. Indeed, final retinal attachment can be achieved in 93.2 to 99.4% of patients with an uncomplicated RRD [1–4].

Over the years, different surgical approaches such as scleral buckling (SB), pars plana vitrectomy (PPV), combined SB and PPV, and pneumatic retinopexy (PR) have been widely proposed according to the retinal break location, posterior vitreous and lens status, and patient age [5]. Although SB is recommended for young phakic patients without posterior vitreous detachment (PVD), PPV still represents the first choice [6].

Despite the treatment, several medium- and long-term complications may occur after successful RRD repair reducing functional recovery and requiring further medical or surgical treatment. Postoperative cystoid macular edema (CME) and epiretinal membrane (ERM) formation represent the most common complications after successful RRD repair [7–11].

The postoperative CME incidence after an RRD repair falls within a wide range of 3–43%, often delaying the visual recovery with a frequency peak of 4–12 weeks [12–16].

Despite postoperative CME multifactorial etiologies, intraocular inflammation plays a crucial role in the development of CME. Indeed, the cytotoxic insult secondary to the postoperative intraocular inflammation causes blood retinal-barrier (BRB) changes, leading to a serous exudation of intraretinal capillaries between the retina's outer plexiform and inner nuclear layers, as well as swelling in retinal Muller cells [7–9, 11].

ERM formation after retinal reattachment surgery is called “macular pucker,” ranging from 4 to 13%. Anatomic alteration due to epiretinal membrane formation after RRD repair often worsens visual acuity and metamorphopsia regardless of the surgical approach [8, 17]. According to Hirakata et al., an increased risk of macular pucker after RRD surgery is significantly associated with preoperative vitreous hemorrhage, multiple retinal breaks, re-detachment, and retinal detachment area regardless of the surgical technique used [13].

This study aims to assess the incidence and risk factors of macular pucker and CME formation after SB and PPV in patients who developed primary rhegmatogenous retinal detachment and to analyze the anatomical and functional outcomes according to the surgical approach used.

## Methods

### Patients

In this monocentric retrospective observational cohort study, we collected pre-and post-operative data from 62 consecutive patients with primary RRD who underwent PPV or SB for primary RRD repair performed between 1 January 2020 and 1 June 2021 at the department of Neuroscience-Ophthalmology of the University of Padova, Italy. In addition, data were retrieved from patients who underwent either SB or PPV ± phacoemulsification and intraocular lens implantation (IOL). The study was carried out with the approval of the Ethics Committee of Ospedale Università Padova and in accordance with the 1976 Declaration of Helsinki and its later amendments. Written informed consent for data collection and analysis of collected data from their medical records was obtained from all patients.

All patients with primary uncomplicated RRD treated by PPV or SB with no history of previous surgical or laser treatment were included in the study. Patients with a history of ocular trauma or previous ocular surgery on the same eye and preoperative conditions that may have been related to CME, such as uveitis, severe non-proliferative or proliferative diabetic retinopathy, recent cataract surgery, retinal vein occlusion or pan-retinal photocoagulation within six months before surgery were excluded. In addition, we further excluded patients with a history of ERM, vitreous hemorrhage (VH); full thickness or lamellar macular holes (MH); tractional-, serous-, and post-traumatic retinal detachments, or any other retinal diseases.

### Examinations

**Fig. 1:**
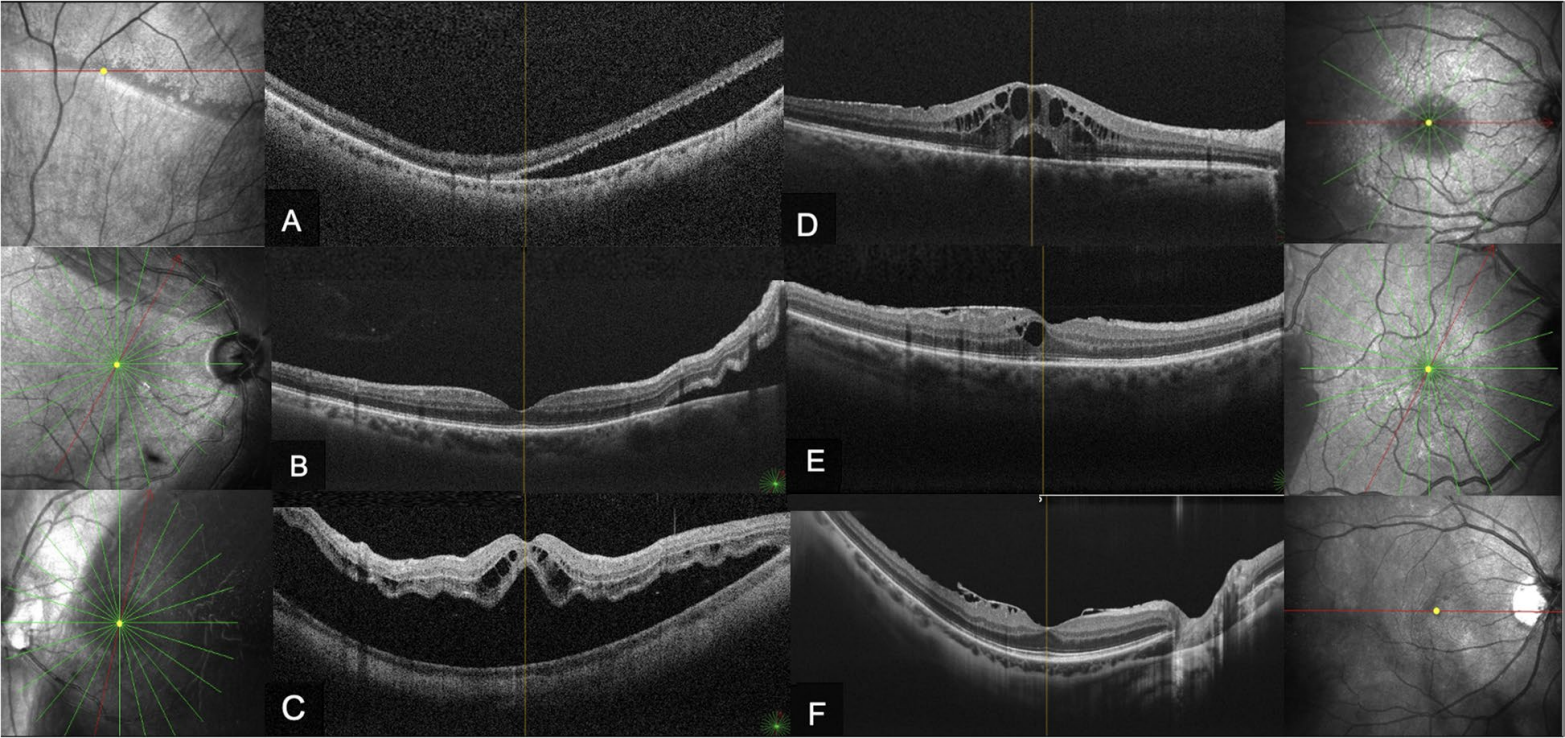
Macula status rhegmatogenous retinal detachment (RRD) patterns **A**: macula-on “properly so-called”; **B**: macula-on “pending foveal detachment”; **C**: macula-off) and postoperative complications (**D**: Cystoid macular edema with subretinal fluid; **E**. Cystoid macular edema with epiretinal membrane; **F**: Epiretinal membrane)

All patients underwent routine examinations before surgery, 3 and 6 months postoperatively, including the measurement of best corrected visual acuity (BCVA) in the logarithm of the minimum angle of resolution (logMAR), the measurement of intraocular pressure (IOP) with a non-contact tonometer, a slit lamp biomicroscopy evaluation and a dilated fundus evaluation using 90 diopter lens. Furthermore, the spectral domain optical coherence tomography (SD-OCT) using the Nidek RS 3000 Advance device (Nidek, Gamagori, Japan) was performed at baseline and every twelve weeks postoperatively, to evaluate the macular changes, including CME and ERM formation (Fig. 1). Central foveal thickness (CFT) was automatically measured by OCT. Two investigators reviewed all OCT images independently (L.M. and R.F.). The diagnosis of CME was made if the macular OCT image showed cystoid spaces within the inner or outer retina with or without subretinal fluid (SRF) between the photoreceptor layer and retinal pigment epithelium (RPE), whereas the ERM was defined as a hyperreflective line above the retinal nerve fibers layer (RNFL) in the macular area and it was staged according to a 4-grade OCT classification [18]. Disagreements were resolved by the consensus of the investigators.

The demographic and preoperative data such as age, gender, lens status (phakic Vs. pseudophakic), IOP, BCVA, and macula status (on or off), as well as postoperative data including BCVA, IOP, fundoscopy at each follow-up visit, OCT imaging to detect the presence of CME and ERM, treatment and response in cases with CME, were recorded, retrieved, and collected. All subjects in this study underwent OCT every twelve weeks after surgery.

Macula status was defined according to the macula and fovea involvement: To be specific, we defined: 1) “macula-off” RRD as an RRD involving the macular area including the fovea; 2) “macula-on RRD” as an RRD that did not involve the macular area. Furthermore, “macula-on RRD” was further divided into two subgroups: 2a) the “properly so-called” RRD, which did not involve the macular area but extended outside the temporal vascular arcades, and 2b) the “pending foveal detachment," which involved the macular area with a still attached fovea. In both types of macula-on RRD, the central vision was intact (Fig. 1).

### Surgical techniques

The surgical approach was undertaken according to RRD characteristics, patient demographics, and clinical parameters. Notably, SB was used in young phakic patients without PVD, high myopic patients (axial length > 29 mm), and RRD associated with either anterior or inferior retinal tears.

All patients underwent the same surgical technique that included the following steps: 1) 360° conjunctival peritomy and isolation of the four rectus muscles, 2) circumferential buckle placement, 3) retinal breaks localization (if possible) with indirect ophthalmoscopy and scleral marking with a diathermy probe 4) Cryotherapy to induce a chorioretinal scar 5) 20% Sulfur-hexafluoride (SF6) intravitreal injection (0.4 ccs) previous subretinal fluid drainage that was performed at the surgeon’s discretion.

PPV was preferred over SB in pseudophakic patients or those with media opacity and posterior breaks that precluded the SB approach. All patients underwent a three-port 23-gauge core, and peripheral PPV performed using a noncontact wide viewing system (Constellation Vision System, Alcon Laboratories, Inc., Fort Worth, TX, USA), followed by endolaser photocoagulation using a curved probe that was performed around the retinal tears and circumferentially (360°). Perfluorocarbon liquid was used to flatten the retina during the procedure. Finally, patients received only 20% SF6 as intraocular tamponade. Phacoemulsification and IOL implantation were performed in phakic patients, whether media opacity or lens bulging did not allow the surgeon to perform surgical maneuvers such as vitreous base shaving adequately. The inner limiting membrane (ILM) peeling was randomly performed in the macula-off RRD group and the macula-on RRD “pending foveal detachment” subgroup.

Due to the lack of macular involvement, ILM peeling was avoided in the macula-on “properly so-called" subgroup. All surgeries were performed by an expert surgeon (R.F.)

### Statistical analysis

All data were collected using the REDCap platform [19], and all statistical analyses were performed using the SPSS version 27 (IBM-SPSS, Chicago, IL, USA).

All variables included in the study were summarized by descriptive statistics techniques. In-depth, qualitative (gender, eye, lens status, macula status, CME, and ERM incidence) variables were expressed as absolute and percentage frequency. As for quantitative variables (age, IOP, BCVA, CFT), we performed the Shapiro–Wilk test to assess their distribution. Then, whether normally distributed, they were described as mean and standard deviation (SD), otherwise, as the median and interquartile range (IQR).

Between groups differences for each parameter considered were assessed, as for qualitative variables, either by the Fisher Exact test or the Chi-square test, with Yates correction, as appropriate. Quantitative variables were instead either evaluated by one-way ANOVA or Student t-test if normally distributed; otherwise, either Mann Whitney U test or Kruskal–Wallis test were applied.

Analysis of Maximum Likelihood Estimates was used to evaluate the risk of CME and ERM in surgical groups, macula status groups (macula-off Vs. macula-on), phacoemulsification plus IOL implantation, and ILM subgroups (yes/no). All correlations were evaluated at 3- and 6 months postoperatively by a linear regression model. In addition, risk factors for CME and ERM were assessed with logistic regression analysis employing the estimation of Odds-Ratios (OR) and their 95% confidence interval. A *p*-value < 0.05 was considered statistically significant.

## Results

### Demographics and clinical data

Sixty-two eyes of sixty-two patients with a mean age of 56.23 ± 13.2 years fulfilled the inclusion criteria and were enrolled in the study. Of these, 20 patients underwent SB and 42 PPV. All patients did not develop any surgical complications during and after surgery, and the retinal reattachment was obtained with a single surgery. Demographic and preoperative clinical data of surgical groups (SB group = 20 eyes, PPV groups = 42 eyes) are shown in Table 1. Overall, SB and PPV groups significantly differed for age (*p* = 0.0005), BCVA (*p* = 0.0002), and macula status (*p* = 0.0001). Therefore, patients who underwent SB were younger, with a better BCVA and a higher percentage of macula-on RRD than patients who underwent PPV. In the SB group, SRF drainage was performed in 9 out of 20 patients (three macula-off, five macula-on “properly so-called,” one macula-on “pending fovea detachment"). In the PPV group, phacoemulsification plus IOL implantation was performed in 30 out of 42 patients (twenty-one macula-off, seven macula-on “properly so-called,” two macula-on “pending foveal detachment"). ILM peeling was performed in 17 patients (15 out of 30 with macula-off and two out of four with macula-on “pending foveal detachment").

**Table 1:** Demographic and preoperative clinical data

Parameters	SB	PPV	Total	*p*
**N. of patients** (eyes)	20	42	62	*-*
**Age** (years) *mean* ± *SD (range)*	46.7 ± 14.9 (17–72)	60.8 ± 9.5 (36–78)	56.23 ± 13.2 (17–78)	*0.0005*
**Gender** (female/male) *number (%)*	24(60) /16(40)	38(45.3) /46(54.)8	31(50)/31(50)	*0.12*
**Eye** (right/left) *number (%)*	6(30) /14 (70)	20(47.6) /22(52.4)	26(41.9) /36(58.1)	*0.24*
**BCVA** (logMAR) *mean* ± *SD (range)*	0.3 ± 0.7 (0–2.8)	1.4 ± 1 (0–3)	1 ± 1 (0–3)	*0.0002*
**IOP** (mmHg) *mean* ± *SD (range)*	16.1 ± 5.3 (8–23)	15.2 ± 4.6 (4–28)	15.8 ± 6.2 (4–28)	*0.32*
**Lens status** P/PP *number (%)*	20 (100) /-	33 (78.6) / 9 (21.4)	53 (80.6) /9 (19.4)	*-*
**Macula status** *number (%)* **ON** properly so-called pending foveal detachment **OFF**	16 (80) 1 (5) 3 (15)	8 (19.1) 4 (9.5) 30 (71.4)	24 (38.7) 5 (8.1) 33 (53.2)	*0.0001*

* Abbreviations SB* *Scleral buckling****,**** PPV* *Pars plana vitrectomy****,**** BCVA * *Best-corrected visual acuity*

*IOP* *Intraocular pressure, P*  *Phakic, PP Pseudophakic, SD Standard deviation*

**Fig. 2:**
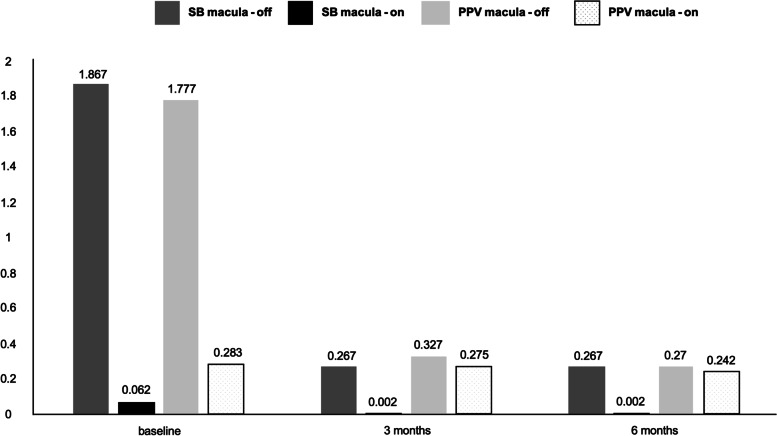
Changes of best-corrected visual acuity (LogMar) in surgical groups (scleral buckling Vs. pars plana vitrectomy) stratified for macula status (macula-on and macula-off)

Figure 2 highlights BCVA changes in SB and PPV groups stratified for macula-off and macula-on status. BCVA improved in both surgical groups (SB and PPV) and macular status groups (macula-on and macula-off), with a significant improvement from baseline to 3- and 6-month follow-ups in the PPV group compared to the SB group (*p* = 0.0003 and *p* = 0.0027, respectively) and in macula-off compared to macula-on groups (*p* = 0.0001 and *p* < 0.001, respectively).

### CME features:

**Fig. 3:**
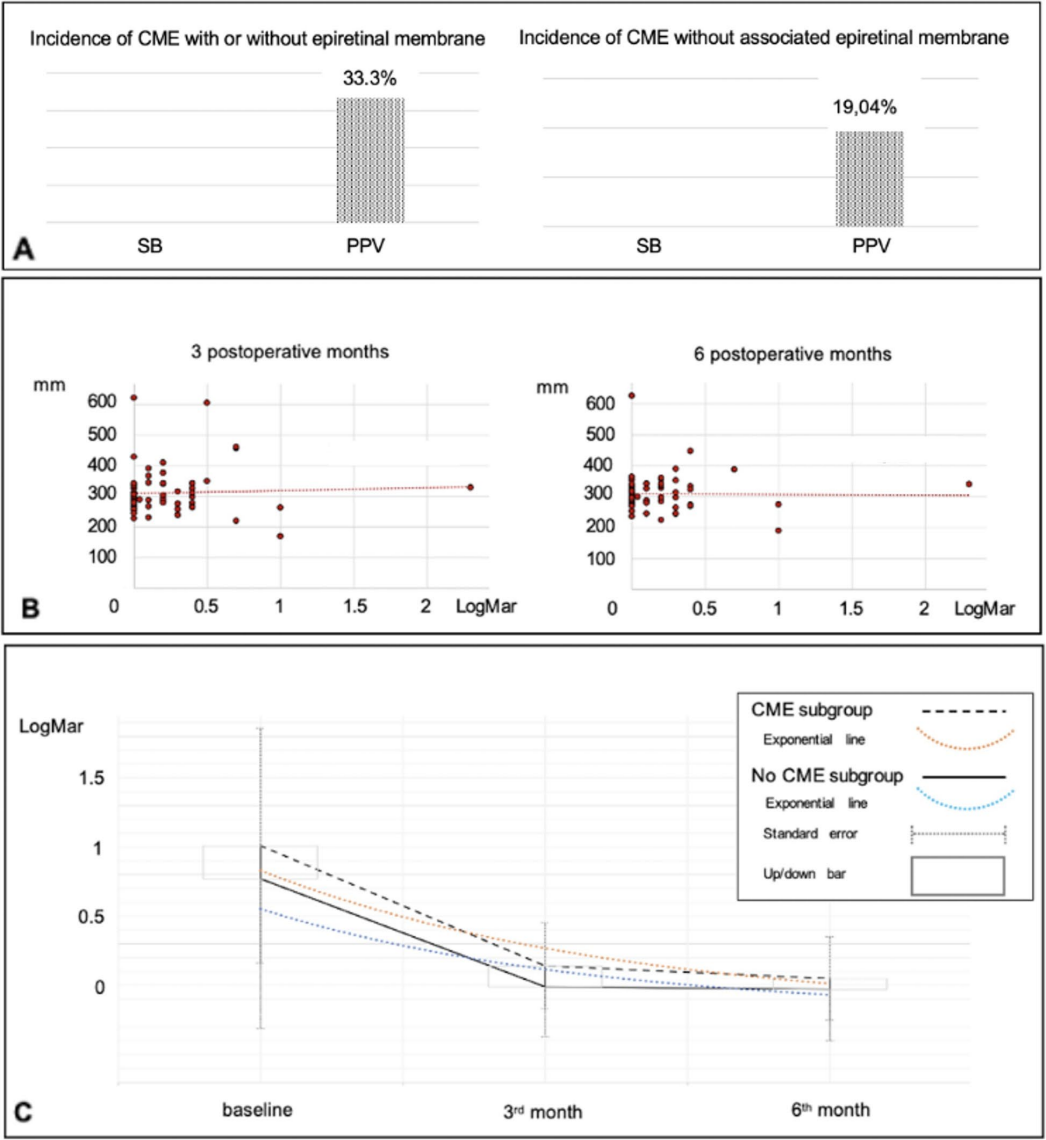
Cystoid Macular Edema (CME) characteristics. **A** Incidence of CME in surgical groups; **B** Relation between best-corrected visual acuity and central foveal thickness; **C** Best-corrected visual acuity in patients with or without CME)

CME occurred in 33.3% (14/42) of the PPV group regardless of the ERM formation. No CME cases were found in the SB group. (*p* = 0.001) (Fig. 3a). The mean onset time was 72.71 ± , 31.5 days, ranging from 24 to 141 days.

**Fig. 4:**
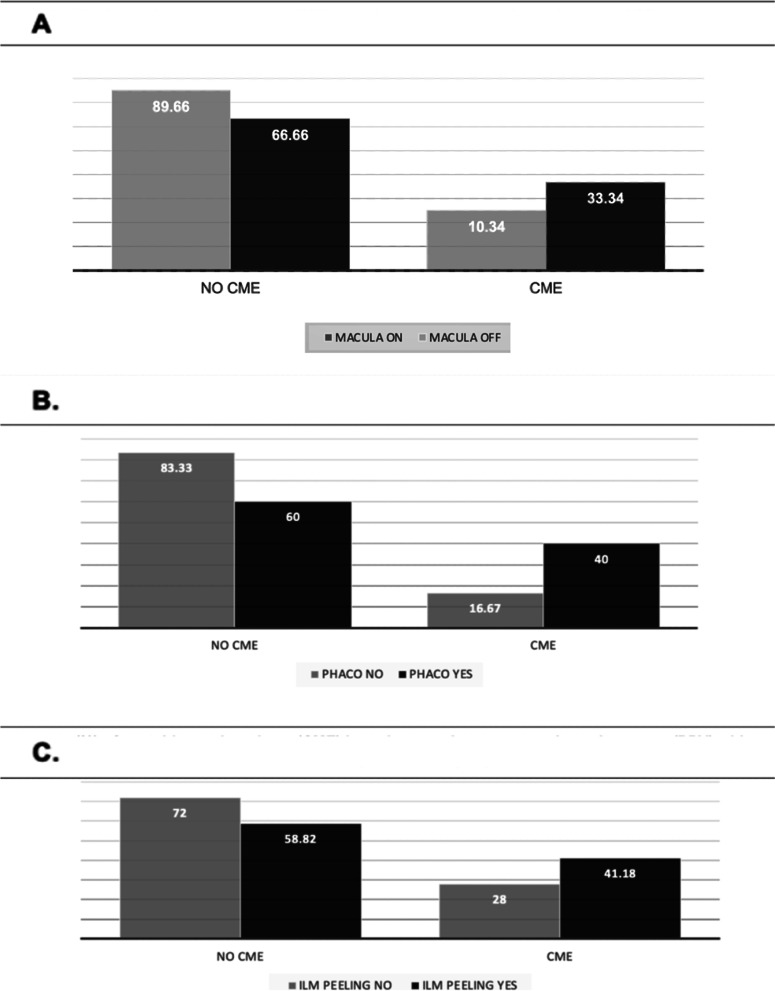
Frequency (%) of cystoid macular edema (CME) in patients with macula-on and macula-off rhegmatogenous retinal detachment (**A**); in patients who underwent pars plana vitrectomy (PPV) with or without phacoemulsification and intraocular (IOL) implantation (**B**); in patients who underwent PPV with or without internal limiting membrane (ILM) peeling (**C**)

The mean 3-month CFT in the CME subgroup was 404.5 ± 106.80 µm (range 263–623 µm), whereas the mean 6-month CFT in the CME subgroup was 364.86 ± 88.2 µm (range 274–626 µm). The mean 3-month CFT in the non-CME subgroup was 285.19 ± 37.8 µm (range 169–367 µm), whereas the mean 6-month CFT in the non-CME subgroup was 292.27 ± 34.9 µm (range 190–352 µm). Therefore, the mean CFT was significantly higher in patients with CME compared to patients without CME three months (*p* < 0.001) and six months postoperatively (p < 0.0001), regardless of the surgical approach (Table 2). Three months postoperatively (highest CME incidence), CFT was significantly inversely correlated to BCVA, i.e., the higher the CFT, the worse the BCVA (R^2^ = 0.0019, *p* < 0.05), whereas six months postoperatively (lowest CME incidence) it was not significantly correlated to BCVA**.** (R^2^ = 0.0002, *p* > 0.05) (Fig. 3b and c). The analysis of maximum likelihood estimates demonstrated that macula-off status increased the risk of CME of OR = 4.3 times compared to macula-on regardless of the procedure (*p* = 0.04) (Fig. 4a). Furthermore, the analysis of maximum likelihood estimates demonstrated that macula-off status increased the risk of CME of OR = 1.73 times compared to macula-on in patients who underwent PPV (*p* = 0.4). Regarding the combined surgical procedures, PPV plus phacoemulsification plus IOL implantation increased the risk of postoperative CME by OR = 3.3 times (*p* = 0.16), and ILM peeling increased the risk of postoperative CME by OR = 1.8 times (*p* = 0.37) (Fig. 4b and c).

**Table 2:** Central foveal thickness (μm) in patients with or without cystoid macular edema

	**3 months**	**6 months**
**CME subgroup** *mean SD range*	404.5 ± 106.8 (263–623) µm	364.86 ± 88.2 (274–626) µm
**NO CME subgroup** *mean SD range*	285.19 ± 37.8 (169–367) µm	292.27 ± 34.9 (190–352) µm
***P-value***	< *0.0001*	< *0.0001*

*Abbreviations CME: Cystoid Macular Edema; SD: Standard Deviation;* µm*: micrometre*

At the end of the follow-up, resolution of CME was observed in 13 out of 14 patients (92.86%). Despite the treatment (indomethacin three times daily up to resolution), CME did not resolve in one patient.

### ERM features:

**Fig. 5:**
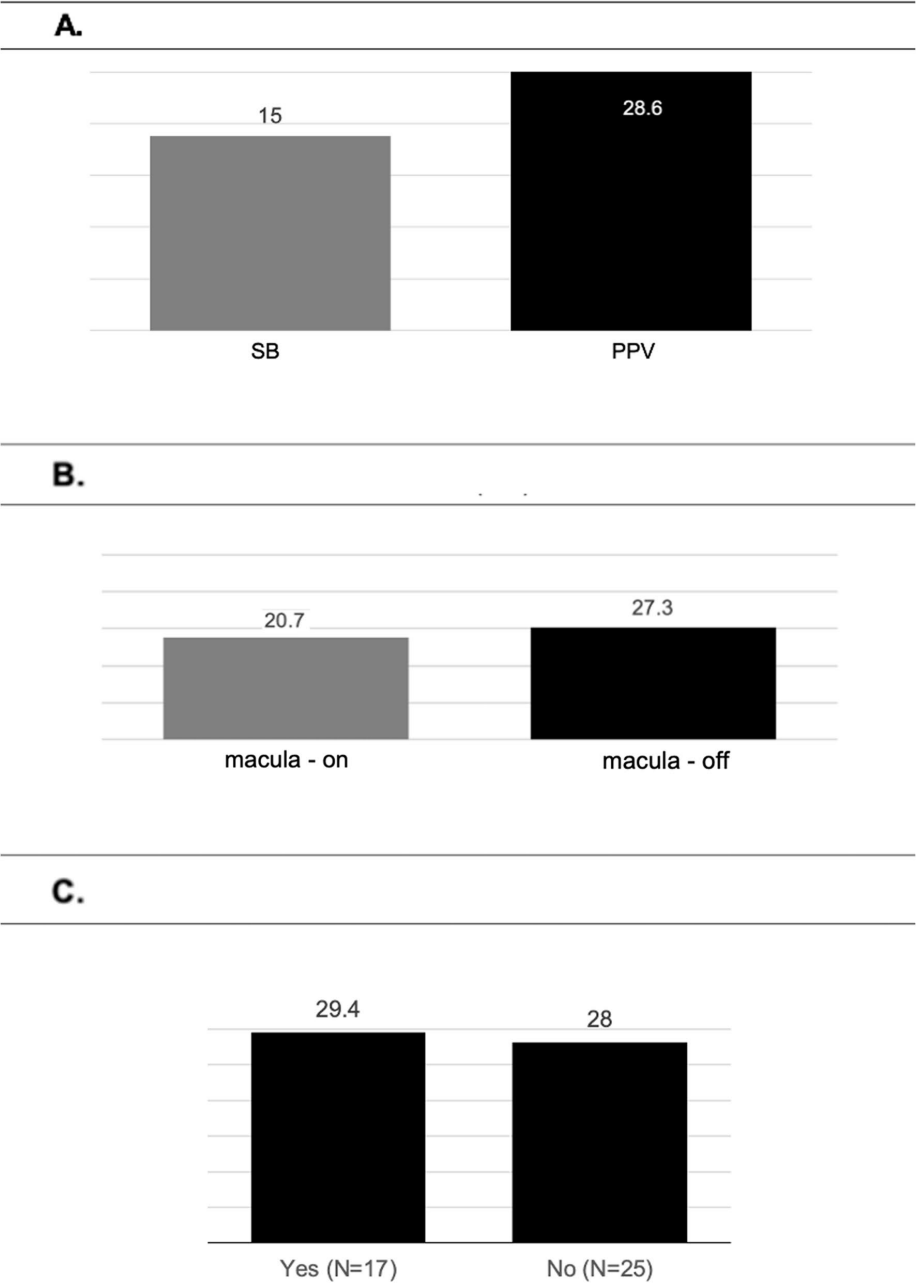
Epiretinal membrane (ERM) frequency (%) in surgical groups (scleral buckling Vs. pars plana vitrectomy) (**A**); in patients affected by macula-on and macula-off rhegmatogenous retinal detachment (RRD) (**B**); in patients who underwent pars plana vitrectomy with and without internal limiting membrane (ILM) peeling (**C**)

Postoperative ERM occurred in both surgical groups (SB = 3 out of 20, 15%, and PPV = 12 out of 42, 28.57%) with no significant differences (*p* = 0.24). (Fig. 5a) No significant relationship was demonstrated between SRF drainage combined with SB and ERM development. Regarding the PPV group, combined phacoemulsification plus IOL implantation did not affect the frequency of postoperative ERM development.

However, ERM occurred in 28% of patients (7 out of 25) who did not undergo ILM peeling and 29.42% (5 out of 17) of the patients who underwent ILM peeling developed ERM (*p* = 0.6) (Fig. 5c). There was no significant relationship between macula status and postoperative ERM occurrence (*p* = 0.54) (Fig. 5b). Six cases of CME were associated with ERM: None of them occurred in the SB group, but only in the PPV group. (6 out of 42, 14.28%). The CME incidence in the PPV group without an associated epiretinal membrane was 19.04% (Fig. 3a). Despite tractional components in 3 out of 20 patients, no CME was associated with ERM in the SB subgroup.

## Discussion

Our study’s main purpose was to evaluate the incidence and risk factors for CME and ERM development after surgery for primary RRD.

Despite several clinical trials’ results, there is no consensus on the surgical management of primary RRD [1, 2, 4, 5], and the treatment choice is often related to the surgeon's experience. Therefore, RRD repair is not often “standardizable.” Notably, there is still no strong current evidence supporting the superiority of one surgical for surgical management of phakic patients with moderately complex primary RRDs [20]. Moreover, different surgical variations have been proposed for SB and PPV. In-depth, different buckles, such as circumferential or segmental buckles, may be used, and the chorioretinal scar may be achieved by either using an endo-laser or cryotherapy. In addition, the trans-scleral fiber-optic-assisted SB is widely used as it permits an easy identification and treatment of retinal breaks using a non-contact wide-angle PPV-style viewing apparatus [21–23]. Similarly, several alternative procedures may be performed during PPV, such as 360° endolaser, lens removal (regardless of its opacity), and ILM peeling [24, 25]. In addition, many surgeons still prefer the PPV/SB procedure because it theoretically offers the advantages of both PPV and SB and has been shown to produce excellent anatomic results [26].

Despite several studies' results, in patients with uncomplicated rhegmatogenous RRD, the most beneficial surgical maneuvers are still controversial. Therefore, we applied restrictive criteria to avoid surgical technique bias and performed the utmost standard procedure. Indeed, PPV was always performed along with a 360° laser and 20% SF6 tamponade use. Nonetheless, ILM peeling (a non-standard surgical procedure for RRD repair) was randomly performed according to macula status. Notably, ILM peeling was only alternatively performed in the macula-OFF group and macula-ON “pending foveal detachment” subgroup. In addition, the SB technique was performed using only the circumferential buckle, cryocoagulation, and 20% SF6 intravitreal injection. Finally, only the SRF drainage was performed according to the surgeon’s choice.

Our results showed significant differences in CME incidence when comparing patients who underwent PPV and SB. Specifically, only 33% of patients who underwent PPV developed a postoperative CME. Previous studies have reported a wide range of incidence rates (5.6–43%) of CME following surgical primary RRD repair [8, 27].

Chatziralli et al. reported a post-PPV and gas tamponade CME incidence of 16.3% [28]. Pole et al. analyzed CME incidence in a sample of 97 patients who underwent SB, PPV, or a combination of both. Despite the study's retrospective nature, CME incidence was 4% after SB, 28% after PPV, and 68% after PPV plus SB [8]. In a case series of 128 patients who underwent SB or PPV or a combination of both for RRD, Gebler et al. reported a CME incidence of 18.7% [17].

Our study detected no CME after SB surgery, whereas a higher incidence of CME was seen in patients who underwent PPV.

Furthermore, according to the CME morphology, only six cases of CME were associated with ERM. Notably, none of them occurred in the SB group but only in the PPV group. (6 out of 42, 14.28%). However, it is challenging to recognize the real etiology of CME due to the different possible coexisting mechanisms. (inflammatory/exudative component Vs. tractional component). Nonetheless, 19.04% of patients who underwent PPV in the absence of ERM developed CME. There was no significant relationship between ILM peeling and postoperative ERM occurrence (*p* = 0.6).

Pole et al. highlighted a significant correlation between CME and ERM after SB for RRD repair. Nonetheless, after the ERM removal, the CME resolution was achieved in only one case, thus stating that CME had an inflammatory/exudative nature rather than a tractional one [8]. In our study, none of the patients who underwent SB developed CME. Therefore, SB may be safer than PPV in achieving surgical RRD repair, consistent with other research that reported a higher risk of CME associated with any ab-Interno macular surgery [29].

The combined procedure of phacoemulsification and the IOL implantation plus PPV (“phacovitretomy”) was associated with approximately threefold higher odds of developing the CME. Nonetheless, the combined phacovitrectomy has several advantages. First, in patients with significant cataracts, a combined procedure in which the surgeon addresses the cataract first optimizes the view and surgical access to the retina, thus improving the visualization for more detailed retinal work [30]. Second, it leads to an overall faster recovery time as PPV can induce lens opacification that is most likely to occur in a reasonably short time, thus affecting postoperative visual recovery [31]. Third, it eases surgical maneuvers reducing the “lens touch” that may lead to increased complication rates in subsequent cataract surgery [32]. Moreover, despite a high risk of “refractive surprise,” many surgeons remove the natural lens in combination with the PPV, regardless of the cataract [33]. Despite these several advantages, the higher risk of CME after combined surgeries cannot be underrated, as the postoperative inflammation can compromise irreversible functional recovery [34].

ILM peeling has become a crucial step in treating various macular diseases during PPV. Representing a scaffold for cell proliferation, ILM removal has been shown to decrease the recurrence rate of idiopathic ERMs, and their development after successful vitrectomy for RRD repair ranges from 6 to 48% [35]. Despite the advantages, it is still a risky procedure that can cause unpredictable macular damage. Indeed, ILM removal is a maneuver that stresses the macular structure and may induce a weakening of the retinal cellular architecture [36, 37].

In our study, we performed ILM peeling in some cases according to the advantages reported in recent studies that highlight the rationale of providing a greater elasticity to the macula during its reattachment [38]. Specifically, we only performed the ILM peeling on macula off and macula-on “pending foveal detachment” but not in macula-on "properly so-called," as in these patients, the macular structure was intact, and ILM peeling could induce a retinal displacement with subsequent metamorphopsia and visual disturbances. Nonetheless, according to our results, the ILM peeling was not significantly associated with postoperative CME.

Despite several research evaluating the risk factors and incidence rate of either ERM or CME after RRD repair using either SB or PPV [20, 28], our article’s main strength is the qualitative and quantitative data analysis that compares the CME and ERM rates after SB and PPV as data comparing their rates between these two surgical groups are limited. Nevertheless, this study has several limitations. First, this was a retrospective study and a non-randomized investigation involving a small sample size. Therefore, our conclusion should be interpreted with caution. Second, we did not consider the retinal tears numbers and their location as a deciding factor for the surgical technique. Third, we randomly performed the ILM peeling on macula-off and macula-on “pending foveal detachment” but not in macula-on "properly so-called." A final drawback of the present study is that 6-month outcomes may not necessarily indicate long-term outcomes, as ERM and CME may arise long after a successful RRD repair. Therefore, further studies with more extended follow-up periods and more significant numbers of randomized patients are recommended.

## Conclusion

The risk of postoperative CME was higher in patients with macula-off RRD than in macula-on RRD, regardless of the surgical technique, and in those with macula-off RRD who underwent PPV. Therefore, the SB would be advisable in patients with RRD sparing the macula. Furthermore, despite having several advantages, the combined phacovitrectomy highly increased the risk of postoperative CME.

## Data Availability

The datasets used and/or analyzed during the current study are available from the corresponding author upon reasonable request.

## Abbreviations

CME: Cystoid macular edema
ERM: Epiretinal membrane
RRD: Rhegmatogenous retinal detachment
SB: Scleral buckling
PPV: Pars plana vitrectomy
PVD: Posterior vitreous detachment
IOL: Intraocular lens
PR: Pneumatic retinopexy
BRB: Blood retinal-barrier
VH: Vitreous hemorrhage
MH: Macular hole
BCVA: Best corrected visual acuity
LogMar: Logarithm of the minimum angle of resolution
SD-OCT: Spectral Domain-Optical Coherence Tomography
CFT: Central foveal thickness
RPE: Retinal pigment epithelium
RNFL: Retinal nerve fibers layer
SF6: Sulfur-hexafluoride
SD: Standard deviation
IQR: Interquartile range
OR: Odds-Ratios
ILM: Inner limiting membrane
SRF: Subretinal fluid
